# Abnormal Vibration Identification of Metro Tunnels on the Basis of the Spatial Correlation of Dynamic Strain from Dense Measurement Points of Distributed Sensing Optical Fibers

**DOI:** 10.3390/s25206266

**Published:** 2025-10-10

**Authors:** Hong Han, Xiaopei Cai, Liang Gao

**Affiliations:** 1 School of Traffic and Transportation, Beijing Jiaotong University, Beijing 100044, China; 2 School of Rail Transportation, Shandong Jiaotong University, Jinan 250300, China; 3 School of Civil Engineering, Beijing Jiaotong University, Beijing 100044, China; xpcai@bjtu.edu.cn (X.C.); lgao@bjtu.edu.cn (L.G.)

**Keywords:** metro reserve, tunnel, abnormal vibration identification, distributed optical sensing technique, identification model

## Abstract

The failure to accurately identify abnormal vibrations in protected metro areas is a serious threat to the operational safety of metro tunnels and trains, and there is currently no suitable method for effectively improving the accuracy of abnormal vibration identification. To address this issue, an accurate method for identifying abnormal vibrations in a metro reserve based on spatially correlated dense measurement points is proposed. First, by arranging distributed optical fibers along the longitudinal length of a tunnel, dynamic strain vibration signals are extracted via phase-sensitive optical time-domain reflectometry analysis, and analysis of variance (ANOVA) and Pearson correlation analysis are used to jointly downscale the dynamic strain features. On this basis, a spatial correlation between the calculated values of the features of the target measurement points to be updated and its adjacent measurement points is constructed, and the spatial correlation credibility of the dynamic strain features between the dense measurement points and the target measurement points to be updated is calculated via quadratic function weighting and kernel density estimation methods. The weights are calculated, and the eigenvalues of the target measurement points are updated on the basis of the correlation credibility weights between the adjacent measurement points. Finally, a support vector machine (SVM) and back propagation (BP) identification model for the eigenvalues of the target measurement points are constructed to identify the dynamic strain eigenvalues of the abnormal vibrations in the underground tunnel. Numerical simulations and an experiment in an actual tunnel verify the effectiveness of the proposed method.

## 1. Introduction

During subway operations, timely and accurate identification of abnormal vibrations in protection zones is crucial to ensure the safe operation of trains and the integrity of tunnel structures [1,2]. Existing safety management technologies and methods for identifying abnormal vibrations face certain limitations, particularly for long and continuously operating subway tunnels, where current technologies can no longer meet practical demands. Therefore, there is an urgent need for an effective method for precisely identifying abnormal vibrations [3,4].

Owing to its exceptional resistance to electromagnetic interference, low power consumption, high sensitivity, and extensive monitoring range, distributed fiber optic sensing (DFOS) technology offers significant advantages for intelligent monitoring in long-distance continuous operational scenarios [5,6,7,8]. Leveraging optical scattering mechanisms—such as Raman, Brillouin, and Rayleigh scattering—this technology achieves centimeter-level spatial resolution with measurement point densities in millions, effectively overcoming the spatial discontinuity inherent in traditional point-based sensor systems [9,10,11,12]. The fully distributed sensing networks built on this technology have been widely deployed across multiple critical infrastructure sectors, including structural health monitoring of railways [13,14], environmental safety assessment [15,16,17,18], integrity management of oil and gas pipelines [19,20], and anomaly detection and localization [21,22,23,24].

Fiber optic sensors can be classified into three categories on the basis of their sensing principles and structural configurations: distributed, quasidistributed, and point-type sensors. Among them, phase-sensitive optical time-domain reflectometry (Φ-OTDR)-based distributed fiber optic sensing systems are characterized by a simple structure, long monitoring range, high localization accuracy, and high sensitivity to disturbances. These attributes make them particularly suitable for long-term monitoring of linear and large-scale engineering structures such as subway tunnels and long-span bridges [25,26,27,28]. Consequently, the effective integration of the long-range, high-density monitoring capabilities of distributed optical fibers with varied structural configurations has emerged as a key research focus.

In terms of engineering applications, Liu Yang’s research team proposed a method for detecting uneven settlement in shield tunnels by combining overall settlement data with a smoothing compensation algorithm. This method uses high-spatial-resolution data from distributed fiber sensing to significantly improve monitoring accuracy [29]. In another study, Marie et al. developed a joint time-frequency analysis framework that incorporates empirical mode decomposition and Hilbert transform for processing distributed fiber vibration signals, contributing to the advancement of nonuniform settlement monitoring techniques [30].

Despite these promising results, Rayleigh scatter-based Φ-OTDR systems remain susceptible to external random vibrations—such as those caused by wind, rain, traffic, and building activities—in practical engineering environments [30]. In addition, signal quality is compromised by multiple noise sources, including thermal noise from photoelectric conversion circuits, analog-to-digital conversion quantization errors, and other electronic noise, exacerbated by multiphysics coupling effects. Experimental data show that in typical subway tunnel monitoring scenarios, the raw signal-to-noise ratio is generally less than 15 dB, which severely impedes effective feature extraction [31]. Therefore, multidimensional noise reduction for data signals acquired in complex engineering environments has become a critical technical challenge that must be addressed to enhance the reliability of distributed fiber optic monitoring systems.

Fiber optic sensing signals are often contaminated by various sources of noise, leading to the problem of a reduced signal-to-noise ratio [32,33,34,35], and many scholars have tried various methods to address the vibration signals collected from Φ-OTDR. Xu CJ used spectral subtraction to reduce the broadband background noise while enhancing the time-frequency features of the oscillation information to extract additional features, and they used a support vector machine (SVM) to differentiate different vibration signals for multievent classification [36]. Huang XL also achieved noise reduction in Φ-OTDR signals by combining the random forest algorithm with a smoothing filter. In time-domain feature extraction, metrics such as the median, mean, short-time energy, and number of times past zero can be obtained, whereas frequency-domain feature extraction can yield features such as the maximum magnitude-to-frequency ratio, frequency-domain amplitude, and energy [37]. Although the existing methods have effectively improved the signal quality through wavelet threshold denoising, spectral subtraction and Wiener filtering, they focus mostly on optimizing the processing of a signal at a single measurement point and fail to fully explore the spatial correlation characteristics of dense sensor arrays, which, to a certain extent, restricts the precise identification and classification effectiveness of external abnormal vibrations.

According to the above analysis, effectively using the information correlations from the dense measurement points of distributed sensing optical fibers to achieve accurate identification of abnormal vibrations is a key problem to be solved. Therefore, a new abnormal vibration identification method for metro protection areas is proposed; this approach employs the longitudinal distribution of distributed optical fibers along underground tunnels and the extraction of dynamic strain signal data. By using the spatial correlation of the extracted dynamic strain from the dense measurement points of the distributed sensing optical fibers, the identification accuracy of the classification model is improved, abnormal vibrations in underground tunnels can be accurately identified, and the safe operation of underground tunnels and trains can be ensured.

## 2. Theory of the Proposed Method

### 2.1. Extraction of Multidimensional Dynamic Strain Features

Owing to the characteristics of the φ-OTDR system, the basic quality of the directly acquired dynamic strain vibration signals is poor, and there is obvious amplitude noise, which has a considerable impact on the subsequent feature calculation. Therefore, before the dynamic strain features from the vibration signals are extracted, it is necessary to preprocess the vibration signals to identify, reject and fit the outliers in the data signals.

Unlike the traditional standard deviation, which is highly influenced by extreme values, the interquartile range method is more robust to complex data such as vibration signals from real tunnels, which are not normally distributed. Moreover, the regression method can utilize information from other variables to predict missing values and produce more accurate results than simple mean or median filling does. In particular, the regression filling process is more suitable for the proposed method in terms of enhancing the eigenvalues of the measurement points because it introduces the characteristics of the frequency domain from the neighboring measurement points; thus, the correlation of the variables between the different measurement points can be preserved as much as possible.

The interquartile range method was selected for obvious single-point noise to determine the outlier identification threshold. The signal amplitudes are sorted in ascending order, the first and third quartiles are calculated, and then, the quartiles calculated via Equations (1) and (2) are used to determine the thresholds for identifying outliers and to identify the outlier data points above the thresholds.

(1) $$y_{\max}=Q_{3}+3\times (Q_{3}-Q_{1})$$

(2) $$y_{\min}=Q_{1}-3\times (Q_{3}-Q_{1})$$

The sharply clustered data points exceeding this threshold are deleted, while the missing data are fitted using regression, which can fully account for the distributional trend of the data itself to ensure that the imputed data have a high degree of confidence [38]. The formula corresponding to this process is expressed as follows:

(3) $$y\prime_{k}=a_{0}+\sum_{i=1}^{m}a_{i}X_{i}+\zeta_{k}$$

where $y_{k}$ is the value that should be captured at the kth missing value, $y\prime_{k}$ is the fitted value calculated at the kth missing value, $a_{0},\;a_{i}$ represents the coefficients of the regression model, and $\zeta_{k}$ is the regression error, *X_i_* represents the signal value at the adjacent spatial position of kth.

To accurately extract the different segments of the vibration signal where abnormal vibration exists, data slicing of the dynamic strain vibration signal is performed by the endpoint detection technique. During this process, the time-domain parametric method is used as a geometric adaptive threshold for endpoint segmentation of the processed vibration signals to obtain data slices of the vibration signals [39] as follows:

The number of sampling points of the expected vibration slice is determined as follows:

(4) $$N_{seg}=T\times F_{s}$$

where $N_{seg}$ is the number of signal points in a slice of the vibration signal data after segmentation has been performed; T is the duration of the extracted expected vibration slice; and $F_{s}$ is the sampling frequency of the signal.

Thus, the threshold $y_{TH}$ is determined as follows:

(5) $$y_{TH}=\xi \times y_{\max}$$

where ξ is the threshold setting parameter; depending on the amplitude characteristics of the signal, its default value is 0.4. $y_{\max}$ is the absolute maximum value of the vibration amplitude of a vibration signal.

The index values of all the vibration data points in the signal whose absolute value of amplitude exceeds $y_{TH}$ are determined, and the set $P_{TH}$ is constructed.

(6) $$P_{TH}=\{k_{1},k_{2},\ldots ,k_{m}\},k_{m}\;\mathrm{is}\;\mathrm{required}\;\mathrm{to}\;\mathrm{satisfy}\;\left|y_{k_{m}}\right|>y_{TH}$$

The starting point $k_{seg(z),begin}$ of the zth vibration signal segment in $P_{TH}$ is determined. $k_{seg(z),begin}$ must be the smallest value of an element in the set that is greater than the end value $k_{seg(z-1),end}$ of the previous segment in the set. The smallest value of an indexed value in the set that is greater than $k_{seg(z),begin}+N_{seg}$ is found, and it represents the end point $k_{seg(z),end}$ of the mth vibration signal segment. Afterward, the signal value whose indexed value satisfies the $\left(k_{seg(z),begin},k_{seg(z),end}\right)$ interval is used as the first vibration signal segment.

Each segment of the vibration signal is determined in turn until the signal data have been fully traversed. For the last vibration signal segment, the number of sampling points of the signal is calculated: if the number of sampling points is greater than $N_{seg}$, the last vibration signal segment is retained; otherwise, it is discarded. During this process, the threshold value $y_{TH}$ is set to ensure the adaptability of endpoint detection, which can prevent failure of the slicing algorithm because of the amplitude. In this case, the coefficient ξ depends on the distribution features of the acquired signal.

After the dynamic strain generation vibration signal is preprocessed, the vibration signal eigenvalues are extracted, and the selection of the eigenvectors determines the processing time of different classifiers to a certain extent. Considering the relative complexity of the signal types studied in the underground tunnel working environment, it is difficult to meet the learning needs of the classifier by using a single eigenvalue for the signals; therefore, the vibration signals are extracted from the multidimensional time and frequency domains with 20 vibration signal eigenvalues [40], which are shown in Table 1.

To effectively identify the features related to abnormal vibrations caused by construction violations, eliminating invalid features from the selected tunnel response features and improving the identification efficiency are essential. A single-factor analysis of variance (ANOVA) method is employed to test whether there are significant differences in the means of a continuous dependent variable (the characteristic values of various abnormal vibration conditions) across different levels of a categorical independent variable (different vibration scenarios). This approach helps to assess the feature differences in abnormal vibrations in subway tunnels across various categories. During the execution of ANOVA for each feature related to construction violations, the differences among the features in different event categories are calculated and output as a χ value. In ANOVA, the χ value indicates the probability of observing the actual differences under the null hypothesis (which states that there are no significant differences in means among the categories). A smaller χ value indicates a greater level of difference among the features across different categories. Generally, a threshold of 0.05 is used, meaning that if the χ value is less than 0.05, that feature significantly aids the classification task.

To further reduce the dimensionality of the selected features, the Pearson correlation coefficient method is used to analyze the correlations produced by abnormal train vibrations. The Pearson correlation coefficient is a statistic used to measure the strength of the linear relationship between two variables. Its value ranges from −1 (perfect negative correlation) to 1 (perfect positive correlation), with values closer to 0 indicating a weaker linear relationship between the two variables. This analysis helps reduce the dimensionality of the selected vibration features, thereby improving the subsequent computational efficiency.

### 2.2. Dynamic Strain Feature Update Method Based on the Spatial Correlation of Distributed Fiber-Optic Dense Measurement Points

#### 2.2.1. Spatial Correlation Between the Dynamic Strain Features Obtained from the Dense Measurement Points of a Distributed Sensing Optical Fiber

In practical engineering applications, φ-OTDR is typically used for the full-length deployment of tunnel segments. However, to verify the effectiveness of the proposed method in the case of a limited fiber deployment length, some of the tunnel monitoring segments were selected for analysis in this study. If the method is still effective under the deployment length limitation, it can significantly improve the accuracy of identifying construction trespass violations regardless of whether the optical fiber is deployed for the full length or not.

Due to the limitations of the optical fiber itself and the influence of environmental noise, even for adjacent or very close measurement points, the collected signals may differ significantly because of factors such as phase variations or noise. Nonetheless, the direct cause of these signal differences between neighboring or close measuring points is the vibration in the zone in which they are located. Therefore, in theory, in the case of a dense measurement point layout, the signals collected from neighboring or close measurement points can consistently reflect some features of the vibration, and there is a similar correlation between these features, which is called the “spatial correlation of dynamic strain features” in this paper.

On the basis of the spatial correlation between the dynamic strain features of the dense measurement points, the feature value can be updated by combining the feature information from the abnormal vibration signals collected at a measurement point with the information from the close measurement points before and after. This process improves the identification accuracy of the features and achieves accurate identification of abnormal vibration signals within the protected section of the metro. This method of information fusion helps improve the early warning capability of abnormal vibration behavior and effectively guarantees the safe operation of railway tunnels.

After the vibration signal is subjected to feature dimensionality reduction, it is fused and updated with the retained vibration signal features. By using this feature, a signal feature updating algorithm based on the spatial correlation of high-density measurement points is constructed via the following process:

For the signals collected at a point P in the monitoring area, a vector $T^{\left(p\right)}$ of the computed eigenvalues is calculated:

(7) $$T^{\left(p\right)}=\left\{\;T_{1}^{\left(p\right)},T_{2}^{\left(p\right)},T_{3}^{\left(p\right)},\ldots ,T_{n-1}^{\left(p\right)},T_{n}^{\left(p\right)}\right\}$$

where $T_{n}^{\left(p\right)}$ denotes the nth feature computed value of the feature vector for the acquired signal at the target measurement point P.

Based on the eigenvalues of the target point P and a total of 2m+1 points before and after the target point, a matrix of eigenvalues for the target point range is formed:

(8) $$T_{total}^{\left(p\right)}=\left\{\left.\begin{array}{l} T^{\left(p-m\right)}\\ T^{\left(p-m+1\right)}\\ \vdots \\ T^{\left(p\right)}\\ \vdots \\ T^{\left(p+m\right)} \end{array}\right\}\right.=\left[\begin{array}{lllll} T_{1}^{\left(p-m\right)} & T_{2}^{\left(p-m\right)} & \ldots & T_{n-1}^{\left(p-m\right)} & T_{n}^{\left(p-m\right)}\\ T_{1}^{\left(p-m+1\right)} & T_{2}^{\left(p-m+1\right)} & \ldots & T_{n-1}^{\left(p-m+1\right)} & T_{n}^{\left(p-m+1\right)}\\ \vdots & \vdots & \ddots & \vdots & \vdots \\ T_{1}^{\left(p\right)} & T_{2}^{\left(p\right)} & \ldots & T_{n-1}^{\left(p\right)} & T_{n}^{\left(p\right)}\\ \vdots & \vdots & \ddots & \vdots & \vdots \\ T_{1}^{\left(p+m\right)} & T_{2}^{\left(p+m\right)} & \ldots & T_{n-1}^{\left(p+m\right)} & T_{n}^{\left(p+m\right)} \end{array}\right]$$

where $T_{total}^{\left(p\right)}$ denotes the matrix of computed values for the range features of the target measurement point P, the dimension is (2m+1)×n.

A vector of distance-dependent confidence weights $w_{d}^{\left(p\right)}$ is also established on the basis of the distance between the target measurement point P and a total of 2m+1 measurement points before and after in the dense set of measurement points:

(9) $$\begin{array}{l} w_{d}^{\left(p\right)}=\left\{w_{d,(p-m)}^{\left(p\right)},w_{d,(p-m+1)}^{\left(p\right)},\ldots ,w_{d,(p-1)}^{\left(p\right)},w_{d,p}^{\left(p\right)},\right.\\ \left.\;\;\;\;\;\;\;\;\;\;\;\;\;\;\;\;\;\;\;w_{d,(p+1)}^{\left(p\right)},\ldots ,w_{d,(p+m-1)}^{\left(p\right)},w_{d,(p+m)}^{\left(p\right)}\right\},m<p \end{array}$$

where $w_{d,(p+m)}^{\left(p\right)}$ is the confidence weighting factor associated with the mth point distance before the target point P and where $w_{d,(p-m)}^{\left(p\right)}$ is the confidence weighting factor associated with the mth point distance after the target point P.

The values of the features for the target measurement point P in the monitoring area are calculated with a total of 2m+1 measurement points before and after, and the probability distribution-related confidence weight coefficients $w_{p,n}^{\left(p\right)}$ for each feature are established via probability density estimation, which in turn first establishes the probability distribution-related confidence weight matrix $w_{p}^{\left(p\right)}$ for the feature as a whole.

(10) $$\begin{array}{l} w_{p,n}^{\left(p\right)}=\left\{w_{p,n,(p-m)}^{\left(p\right)},w_{p,n,(p-m+1)}^{\left(p\right)},\ldots ,w_{p,n,(p-1)}^{\left(p\right)},\right.w_{p,n,p}^{\left(p\right)},\\ \;\;\;\;\;\;\;\;\;\;\;\;\;\;\;\;\;\;\left.w_{p,n,(p+1)}^{\left(p\right)},\ldots ,w_{p,n,(p+m-1)}^{\left(p\right)},w_{p,n,(p+m)}^{\left(p\right)}\right\},\;\;m<p \end{array}$$

where $w_{p,n}^{\left(p\right)}$ represents the vector of the confidence weights associated with the probability distribution of the nth feature of the 2m+1 measurement point in the total number of measurement points before and after the target measurement point P with dimension 1×(2m+1)×1. $w_{p,n,(p+m)}^{\left(p\right)}$ denotes the coefficient of the confidence weights associated with the probability distribution of the nth feature of the mth measurement point before the target measurement point P, and $w_{p,n,(p-m)}^{\left(p\right)}$ denotes the coefficient of the confidence weights associated with the probability distribution of the nth feature of the mth measurement point after the target measurement point P.

The combined weights at the measurement points before and after each feature are calculated by considering both the distance-related confidence weights and the probability distribution-related confidence weights, and then, they are normalized so that they can be used to update the calculated values of the features via the following process:

For the nth feature before and after the target measurement point P, the Hadamard product of the distance-related confidence weight vector and the probability distribution-related confidence weight vector is calculated and normalized to obtain the normalized composite weight vector for the nth feature before and after the target measurement point P. First:

(11) $$\begin{array}{l} {\tilde{w}}_{n}^{\left(p\right)}=w_{d}^{\left(p\right)}⊗w_{p,n}^{\left(p\right)}\\ \;\;\;\;\;\;\;\;\;\;\;\;\;\;\;\;\;\;\;\;\;\;\;=\left\{{\tilde{w}}_{n,(p-m)}^{\left(p\right)}\right.,{\tilde{w}}_{n,(p-m+1)}^{\left(p\right)},\ldots ,{\tilde{w}}_{n,p}^{\left(p\right)},\ldots ,{\tilde{w}}_{n,(p+m-1)}^{\left(p\right)},\left.{\tilde{w}}_{n,(p+m)}^{\left(p\right)}\right\} \end{array}$$

where each element is calculated as follows:

(12) $${\tilde{w}}_{n,(q)}^{\left(p\right)}=w_{d,(q)}^{\left(p\right)}\times w_{p,n,(q)}^{\left(p\right)},\;\;\;\;\;\;\;\;\;q\in [(p-m),(p+m)]$$

where ${\tilde{w}}_{n}^{\left(p\right)}$ denotes the composite weight vector of the nth feature before and after the target measurement point P, ⊗ denotes the Hadamard product calculation, and the composite weight vector is normalized to obtain the normalized composite weight vector:

(13) $$w_{n}^{\left(p\right)}=\left\{w_{n,(p-m)}^{\left(p\right)}\right.,w_{n,(p-m+1)}^{\left(p\right)},\ldots ,w_{n,p}^{\left(p\right)},\ldots ,w_{n,(p+m-1)}^{\left(p\right)},\left.w_{n,(p+m)}^{\left(p\right)}\right\}$$

where $w_{n}^{\left(p\right)}$ is the standardized composite weight vector of the nth feature before and after the target measurement point P.

By using the calculated values of the features from the tunnel target measurement point P and the acquired signals from a total of 2m+1 measurement points, the calculated values of the features from the tunnel measurement point P are updated in the following process:

The normalized composite weight vector of each feature before and after the target measurement point P is calculated in turn according to the previous steps to form the normalized composite weight matrix of the features of the target measurement point P:

(14) $$w={\left\{\left.\begin{array}{l} w_{1}^{\left(p\right)}\\ w_{2}^{\left(p\right)}\\ \vdots \\ w_{n-1}^{\left(p\right)}\\ w_{n}^{\left(p\right)} \end{array}\right\}\right.}^{T}=\left[\begin{array}{lllll} w_{1,(p-m)}^{\left(p\right)} & w_{2,(p-m)}^{\left(p\right)} & \ldots & w_{\left(n-1),(p-m\right)}^{\left(p\right)} & w_{n,(p-m)}^{\left(p\right)}\\ w_{1,(p-m+1)}^{\left(p\right)} & w_{2,(p-m+1)}^{\left(p\right)} & \ldots & w_{\left(n-1),(p-m+1\right)}^{\left(p\right)} & w_{n,(p-m+1)}^{\left(p\right)}\\ \vdots & \vdots & \ddots & \vdots & \vdots \\ w_{1,(p)}^{\left(p\right)} & w_{2,(p)}^{\left(p\right)} & \ldots & w_{\left(n-1),(p\right)}^{\left(p\right)} & w_{n,(p)}^{\left(p\right)}\\ \vdots & \vdots & \ddots & \vdots & \vdots \\ w_{1,(p+m)}^{\left(p\right)} & w_{2,(p+m)}^{\left(p\right)} & \ldots & w_{\left(n-1),(p+m\right)}^{\left(p\right)} & w_{n,(p+m)}^{\left(p\right)} \end{array}\right]$$

The updated vector $T^{\left(p\right)}$ for the nth feature calculated value vector ${\tilde{T}}^{\left(p\right)}$ from the target measurement point P is represented as follows:

(15) $$\begin{array}{c} {\tilde{T}}^{\left(p\right)}=\left\{\left.{\tilde{T}}_{1}^{\left(p\right)},{\tilde{T}}_{2}^{\left(p\right)},\ldots ,{\tilde{T}}_{n-1}^{\left(p\right)},{\tilde{T}}_{n}^{\left(p\right)}\right\}\right.\\ \;\;\;\;\;\;\;\;\;\;\;\;\;\;\;\;\;\;\;\;\;\;\;\;\;\;\;\;\;\;\;\;\;\;\;\;\;\;=\left\{\left.w_{1}^{\left(p\right)}T_{1}^{\left(p\right)},w_{2}^{\left(p\right)}T_{2}^{\left(p\right)},\ldots ,w_{n-1}^{\left(p\right)}T_{n-1}^{\left(p\right)},w_{n}^{\left(p\right)}T_{n}^{\left(p\right)}\right\}\right.\\ \;\;\;\;\;\;\;\;\;\;\;\;\;\;\;\;\;\;\;\;\;\;\;\;\;\;\;\;\;\;\;\;\;\;\;\;\;\;=E(w\circ T_{total}^{\left(p\right)}) \end{array}$$

where ${\tilde{T}}_{n}^{\left(p\right)}$ is the updated value for the calculated value of the nth feature at measurement point P, ${\tilde{T}}^{\left(p\right)}$ is the vector consisting of the updated values of the nth feature calculation values for measurement point P, and E is the unit matrix of dimension n.

Thus, the correlation property between dense measuring points can be used to update the calculated value of the feature of the target measuring point P. The updated feature contains the feature information of the neighboring measuring points, so it can reduce the misjudgment of abnormal vibration detection and thus improve the detection accuracy.

#### 2.2.2. Updating the Dynamic Strain Features on the Basis of the Spatial Correlation Confidence Weight Assignment

Because the different distances between the target measurement point and the near-neighbor measurement points lead to differences in the spatial correlation, to update the calculated values of the features accurately between the dense measurement points, a quadratic function weight assignment method is adopted to calculate the weight of the correlation credibility of the distances between the near-neighbor measurement points.

The distance relationship between the different points of the φ-OTDR linearly increases, and it is assumed that as the distance increases, the confidence of the point P when updating the target point decreases according to a quadratic trend; a quadratic function is used to represent this decay:

(16) $${\tilde{w}}_{d,q}^{\left(p\right)}=-a{\left(q-p\right)}^{2}+w_{\max}$$

where ${\tilde{w}}_{d,q}^{\left(p\right)}$ denotes the confidence of the qth measurement point of the tunnel φ-OTDR when updating the target measurement point P. a denotes the confidence attenuation coefficient, which is expressed as $a=-1/{\left(2m\right)}^{2}$, and $w_{\max}$ denotes the confidence of the measurement point in terms of updating its own information.

The confidence level of each measurement point when updating the information of the target measurement point P is calculated, and the weights are normalized so that the sum of all weights is 1, forming a confidence weight vector related to the distance of the measurement points before and after the tunnel target measurement point p:

(17) $$w_{d,(q)}^{\left(p\right)}=\frac{{\tilde{w}}_{d,(q)}^{\left(p\right)}}{\sum_{i=p-m}^{p+m}{\tilde{w}}_{d,(i)}^{\left(p\right)}},\;\;\;\;\;\;\;\left\{\begin{array}{c} i=(p-m),\ldots ,p,\ldots ,(p+m)\\ q\in [(p-m),(p+m)] \end{array}\right.$$

where $w_{d,q}^{\left(p\right)}$ denotes the confidence weight of the qth measurement point when updating the target measurement point P, which is expressed as follows:

(18) $$\begin{array}{l} w_{d}^{\left(p\right)}=\left\{w_{d,(p-m)}^{\left(p\right)},w_{d,(p-m+1)}^{\left(p\right)},\ldots ,w_{d,(p-1)}^{\left(p\right)},w_{d,p}^{\left(p\right)},\right.\\ \left.\;\;\;\;\;\;\;\;\;\;\;\;\;\;\;\;\;\;\;w_{d,(p+1)}^{\left(p\right)},\ldots ,w_{d,(p+m-1)}^{\left(p\right)},w_{d,(p+m)}^{\left(p\right)}\right\},m<p \end{array}$$

where $w_{d}^{\left(p\right)}$ denotes the vector of distance-related confidence weights from the measurement points before and after the target measurement point P.

The kernel density estimation method is used to calculate the confidence weights for the correlation of the probability distribution for the tunnel vibration features, and the specific steps are as follows: the feature calculation values of all features of the tunnel target measurement point P and a total of 2m+1 measurement points are calculated.

Kernel density estimation is performed by computing the kernel and extracting the probability density function of the calculated value of the feature. The probability distributions of the calculated values of the feature before and after the target measurement point P are described via the following formula:

(19) $$K(x)=\left\{\begin{array}{c} \frac{3}{4}(1-x^{2}),\left|x\right|\leq 1\\ \;\;\;\;\;\;\;\;0\;\;\;\;\;\;\;\;\;,\;\;\;\;\;else \end{array}\right.$$

where K(x) denotes the kernel function of the Epanechnikov kernel.

The probability of occurrence of the calculated value for the feature between the target measurement point P and the total of 2m+1 measurement points appearing before and after is calculated, and this value is then used as the probability distribution-related credibility weight coefficient. The distance-related credibility weight coefficients of the q measurement point near the tunnel φ-OTDR target measurement point p are calculated as follows:

(20) $$w_{p,n,q}^{\left(p\right)}=f_{n}(T_{n}^{\left(q\right)})$$

where $w_{p,n,q}^{\left(p\right)}$ denotes the probability distribution-related confidence weight coefficient for the calculated value of the nth feature at the qth measurement point near the target measurement point P.

The confidence weights associated with the probability distribution of the totality of the established features are as follows:

(21) $$w_{p}^{\left(p\right)}=\left.\left\{\begin{array}{c} w_{p,1}^{\left(p\right)}\\ w_{p,2}^{\left(p\right)}\\ w_{p,3}^{\left(p\right)}\\ \;\;\;\;\;\vdots \;\;\;\;\\ w_{p,(n-1)}^{\left(p\right)}\\ w_{p,n}^{\left(p\right)} \end{array}\right.\right\}$$

where:

(22) $$\begin{array}{l} w_{p,n}^{\left(p\right)}=\left\{w_{p,n,(p-m)}^{\left(p\right)},w_{p,n,(p-m+1)}^{\left(p\right)},\ldots ,w_{p,n,(p-1)}^{\left(p\right)},\right.w_{p,n,p}^{\left(p\right)},\\ \;\;\;\;\;\;\;\;\;\;\;\;\;\;\;\;\;\;\;\;\left.w_{p,n,(p+1)}^{\left(p\right)},\ldots ,w_{p,n,(p+m-1)}^{\left(p\right)},w_{p,n,(p+m)}^{\left(p\right)}\right\},\;\;m<p \end{array}$$

As a result, the probability distribution-related confidence weight $w_{p}^{\left(p\right)}$ for the feature totality is established to characterize the probability distribution-related confidence weights for the feature calculation values of the measurement points before and after the target measurement point P, thus updating the feature calculation values of the signals collected from the target measurement point P of the tunnel φ-OTDR and constructing the classification and identification model on the basis of the updated feature calculation values to identify the vibration caused by the abnormal vibration outside the tunnel.

### 2.3. SVM and BP Classification Model Eigenvalue Identification for Abnormal Vibrations

After the spatial correlation relationship of the dense measurement point is used to update the target measurement point eigenvalues, the updated eigenvalues can better express abnormal vibration, but the updated eigenvalues cannot be directly identified accurately. It is therefore necessary to construct a classification model to identify the eigenvalues related to abnormal vibrations. This paper describes the use of the SVM and BP classification models, which are employed to identify abnormal vibrations. The identification effects of the two classification models are compared and analyzed by numerical simulation and real engineering examples.

The SVM classification model is employed as the normal vibration identification model, where the training set of dynamic strain eigenvalues for the normal operation of metro trains is as follows:

(23) $$Z=\left\{\left(x_{1},y_{1}\right),\ldots \left(x_{l},y_{l}\right)\right\}\in {\left(X\times Y\right)}^{l}$$

where $x_{t}\in X=R^{n},y_{t}\in Y=\left\{-1,1\right\}(t=1,2,\ldots ,l)$ and where $x_{l}$ is the dynamic strain eigenvector. The appropriate kernel function $K(x_{t},x_{j})$ and parameter penalty factor C are chosen and constructed to solve the optimization problem as follows:

(24) $$\min\left[\frac{1}{2}\sum_{t=1}^{j}\sum_{t=1}^{l}y_{t}y_{j}\alpha_{t}\alpha_{j}K(x_{t},y_{j})-\sum_{j=1}^{l}\alpha_{j}\right]$$

The optimal solution is then obtained:

(25) $$\alpha^{*}={\left(\alpha_{1}^{*},\alpha_{2}^{*},\alpha_{l}^{*}\right)}^{T}$$

(26) $$b=y-\sum_{t=1}^{l}y_{t}\alpha_{t}^{*}K\left(x_{t}-x_{j}\right)$$

The decision function is constructed as:

(27) $$f\left(x\right)=\mathrm{sgn}\left(\sum_{t=1}^{l}\alpha_{t}^{*}y_{t}K(x,x_{t})+b^{*}\right)$$

The BP classification model is employed as the identification model for abnormal vibrations. The BP neural network is a type of artificial neural network used for abnormal identification and processing, and due to the BP classification model’s high-efficiency feature learning ability, it can better handle abnormal signals in complex environments. The BP algorithm uses a gradient descent strategy to adjust the parameters by accepting the modified vibration feature samples from the hidden layer to the hidden layer as input and using the sigmoid as the output layer to identify abnormal vibration features. The input layer is calculated as follows:

(28) $$a^{\left(r\right)}=f(W^{\left(r\right)}a^{\left(r-e\right)}+b^{\left(r\right)})$$

where $a^{\left(r\right)}$ is the activation value of the neuron in layer r (output), $W^{\left(r\right)}$ is the weight matrix from layer r−1 to layer r, $a^{\left(r-e\right)}$ is the activation value of r−1 (input), $b^{\left(r\right)}$ is the bias vector of the first layer r, and f(·) is the activation function.

The output layer of the BP classification model is expressed as follows:

(29) $$\overset{\wedge }{y}=\sigma (Z^{\left(r\right)})=\frac{1}{1+e^{-z^{\left(r\right)}}}$$

where $\overset{\wedge }{y}$ represents the predicted probability distribution of the output, $Z^{\left(r\right)}$ represents the weighted output from the output layer, and σ(·) represents the sigmoid function with output values between [0,1].

The cross-entropy loss function L is used to quantify the difference between the predicted and true features as follows:

(30) $$L=-\sum_{i=1}^{K}y_{i}\ln{\overset{\wedge }{y}}_{i}$$

where $y_{i}$ represents the true label code, ${\overset{\wedge }{y}}_{i}$ represents the probability of class i being predicted by the model, and K represents the total number of categories.

By employing the above steps, the SVM or BP classification model for the dynamic strain feature calculation value under normal metro train operating conditions can be obtained, the updated abnormal vibration feature calculation value can be substituted into the constructed classification model, and the classification results can be output, thus accurately identifying abnormal vibrations.

To judge the identification accuracy of the model, performance metrics such as the identification model accuracy are used to evaluate the identification accuracy. These performance metrics are calculated on the basis of the true positives (*TP*s), false negatives (*FN*s), false positives (*FP*s) and true negatives (*TN*s) in an SVM confusion matrix with the following mathematical expressions:

(31) $$accuracy=\frac{TP+TN}{TP+FP+TN+FN}$$

### 2.4. Procedure of the Proposed Method

**Figure 1:**
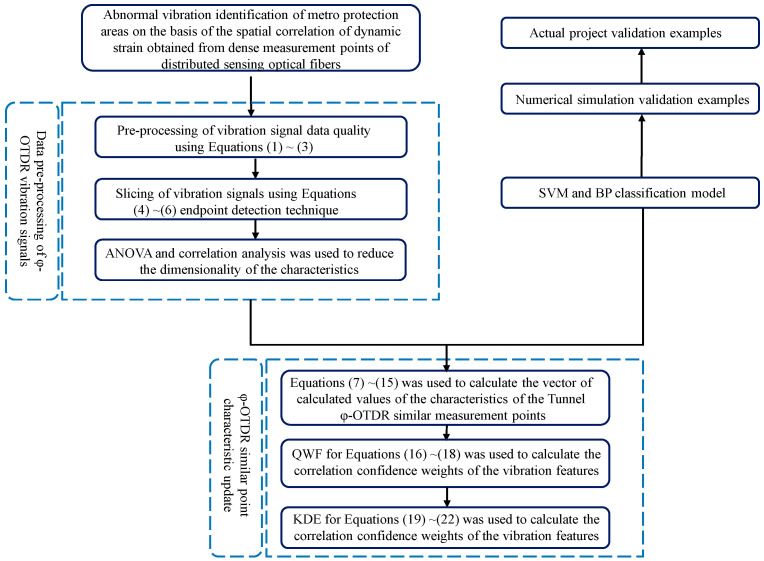
Flow chart of abnormal vibration identification in metro protection areas on the basis of the spatial correlation of dynamic strain obtained from dense measurement points of distributed sensing optical fibers.

The flowchart of the method for identifying abnormal vibrations in metro protected areas on the basis of the spatial correlation of dynamic strain at dense measurement points is summarized in Figure 1.

## 3. Numerical Simulation Examples

### 3.1. Numerical Simulation of the Abnormal Vibration of the Metro Protection Area

Given the strong inhomogeneity of the ground medium in which the tunnel structure is located, the dynamic uncertainty of the surrounding rock-support system’s boundary conditions, and the multi-physical field coupling characteristics of the vibration wave propagation in the viscoelastic-plastic medium, the proposed method was validated by constructing a three-dimensional refined finite element model to numerically simulate the response of the tunnel structure under abnormal vibration of the tunnel periphery to validate the theoretical feasibility of the proposed method in complex geologic environments.

**Figure 2:**
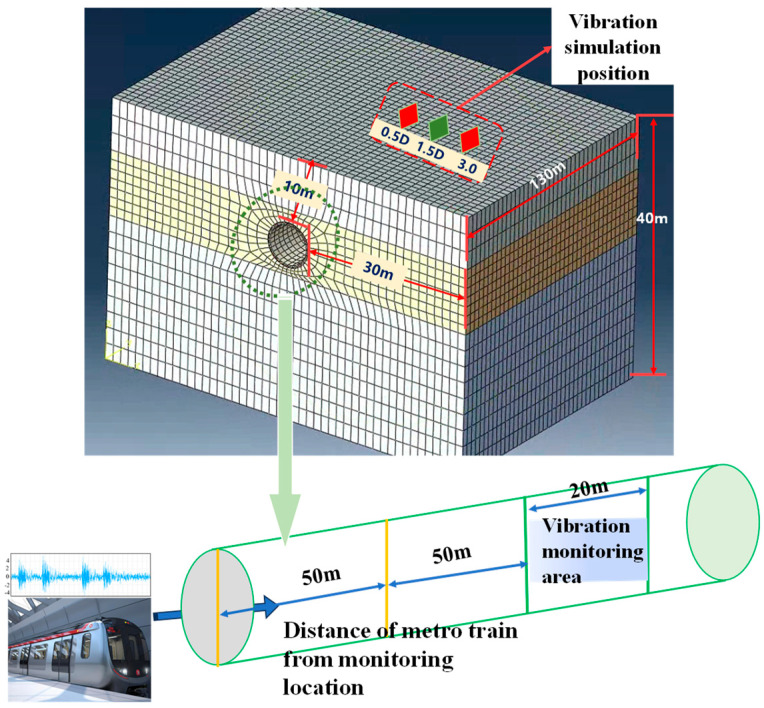
Numerical model of abnormal tunnel vibration.

The ABAQUS 2022 finite element software was used to construct a three-dimensional finite element model of the underground tunnel interval. Based on the boundary effect, and to improve the computational efficiency of the model, the distance between the boundary of the model and the centerline of the tunnel was set to 3–5 times the size of the structure [40,41]. The inner diameter of the tunnel interval lining pipe sheet was 6.1 m, the outer diameter was 6.8 m, and the thickness of the pipe sheet was 0.35 m. Therefore, the finite element model was constructed with 3–5 times the boundary of the tunnel and the centerline of the tunnel. Therefore, the height of the boundary of the finite element model was 40 m, and the top of the tunnel was 10 m from the ground and 30 m on each side of the tunnel centerline. The longitudinal tunnel lining and soil body were 8-node solid units (C3D8R), and the soil body was modeled by the Moore-Cullen intrinsic model. This section of the subway tunnel began operation in 2021, and the tunnel is located in a soil region with top-to-bottom silty clay, clay, and pebble gravel. The specific geotechnical parameters are shown in Table 2, and the construction of the finite element model is shown in Figure 2.

In practice, the strong vibration generated by underground train operation will be coupled with the vibration signals of illegal construction, and this mixed vibration effect will constitute a significant interference to the identification and classification model, leading to its difficulty in accurately separating and identifying the target signals. First, underground train vibration caused by the tunnel structural response needs to be researched. A section of 20 m in the center of the tunnel was designated as the monitoring area, and the train vibration signals at distances of 40 m, 20 m, and 0 m from the monitoring area were selected as the research objects. Based on the vibration parameter conversion algorithm, the collected vibration signals representing the response of the tunnel structure to the actual train operation were converted into strain data [42,43], which were then converted into displacement data by calculating the structural settlement and deformation of shield tunnels based on the high-density measuring point strain [24,44]. The displacement data were applied to the tunnel elevation arch finite element model [45], and the signal features of the monitoring area of the tunnel were extracted according to the vibration features to construct the SVM classification model. The SVM and BP classification models were established on the basis of the extracted vibration features, and the BP classification model was used as the basic identification model for subsequent abnormal vibrations.

**Figure 3:**
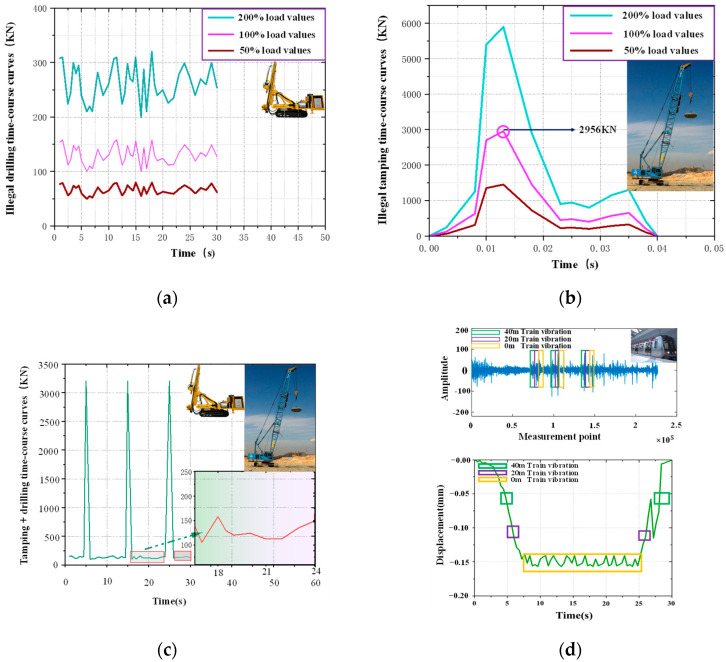
Curves of different loading operations: (**a**) Vibration load curves for drilling operations; (**b**) Vibration load curves for tamping operations. (**c**) Vibration load curves for drilling + tamping operations; (**d**) Dynamic strain signals and load curves of metro trains.

There are a wide variety of abnormal vibration operations around metro tunnels, among which drilling and ramming operations are characterized by small response times, high hazards and hidden work. Once a drilling operation and tamping operation occur to a large extent around a metro tunnel, the tunnel structure will be irreversibly damaged or even destroyed in a relatively short period. The drilling operation (DO), tamping operation (TO), drilling + tamping operation (DTO), and noninterference (NI) were simulated in the model. The signals from the illegal tamping operation around the tunnel were simulated as peripheral vibrations by using the load measurement results from G.G. Goble et al. [20], the signals collected from a typical drilling site were simulated as continuous load vibration after discretization, and the illegal tamping vibration and drilling vibration were combined as the DTO for simulation. Moreover, to verify the effectiveness of the proposed algorithm in terms of identifying different working conditions under different load intensities, 50%, 100% and 200% of the load intensity of normal working conditions were used for the simulation in addition to the vibration signals from an underground train on Jinan Railway Transit Line 2, which were converted into a time series of load curves as the fourth working condition applied to the different locations along the monitoring area [24,45]. The load time series curves of the four working conditions with different intensities are shown in Figure 3.

**Figure 4:**
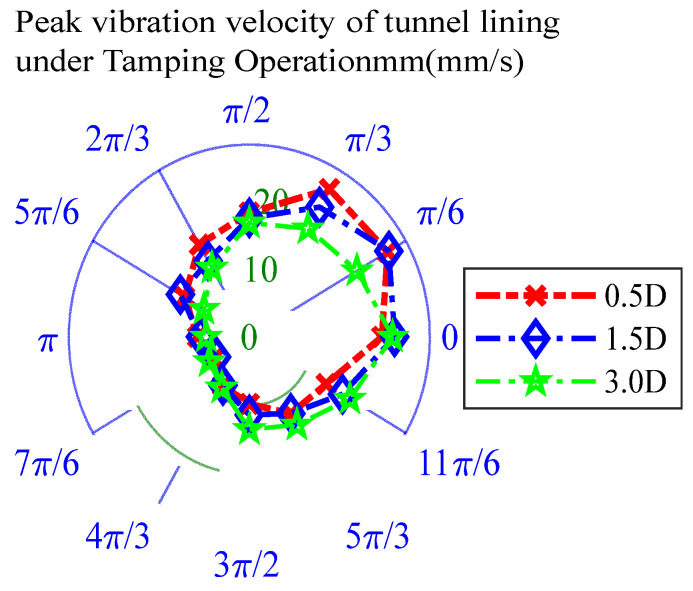
Tunnel response to vibration at different locations.

To determine the most disadvantageous location in the tunnel outside the influence of abnormal vibration, first, the vibration signal of the 100% intensity tamping operation was used as the input point, and the simulation was carried out at horizontal distances of 0.5D, 1.5D and 3.0D Simultaneously, the time–history curve of the vibration velocity at each monitoring point during the vibration operation was extracted, the peak vibration velocity at each point was obtained, and calculate the total vibration velocity, and the results are shown in Figure 4 and Table 3. The effect of vibration loads was most significant at location 1.5D, which was selected as the most unfavorable location for the stressed structure for subsequent load simulations.

### 3.2. Reduction in the Dimensionality of the Dynamic Strain Characteristics Obtained from the Dense Measurement Points of the Distributed Sensing Optical Fiber

**Figure 5:**
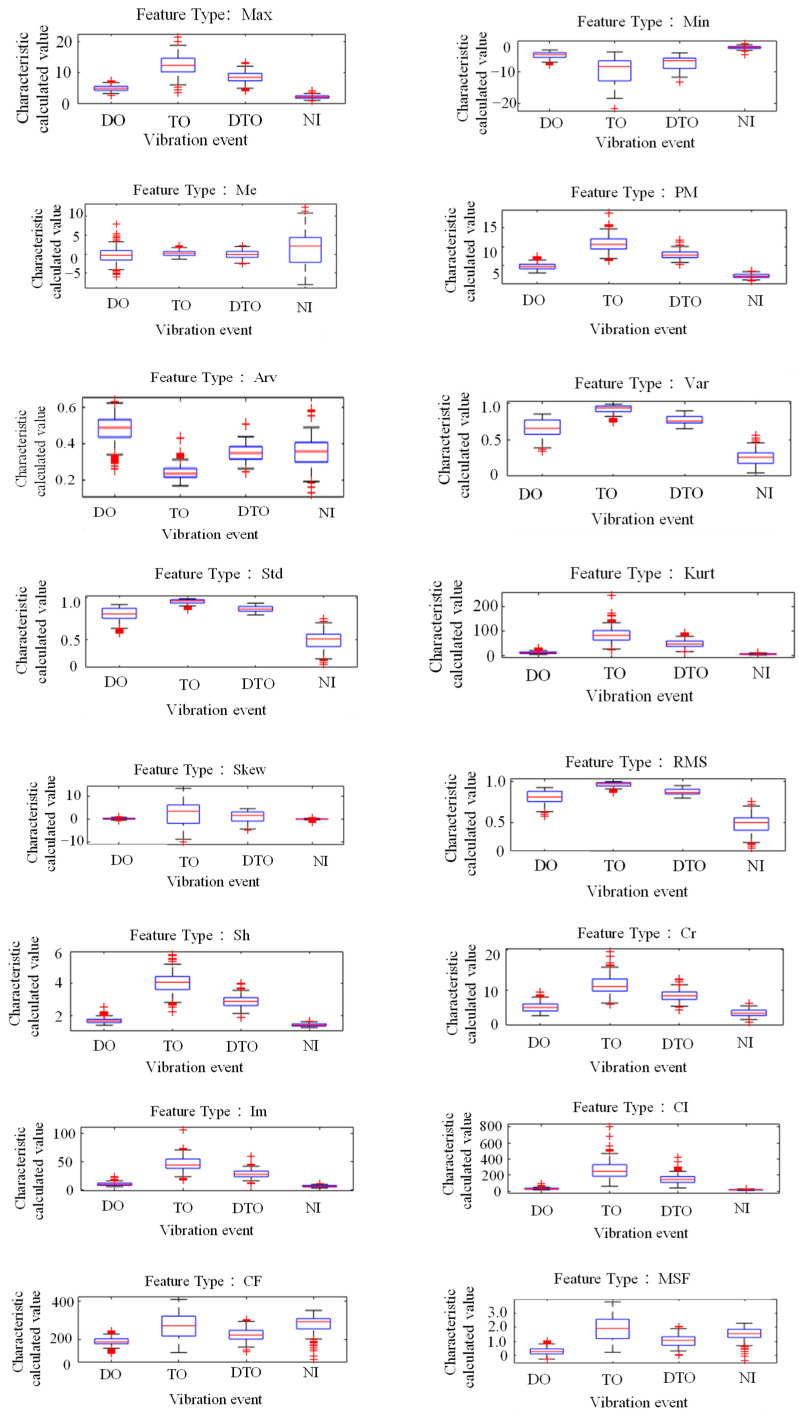

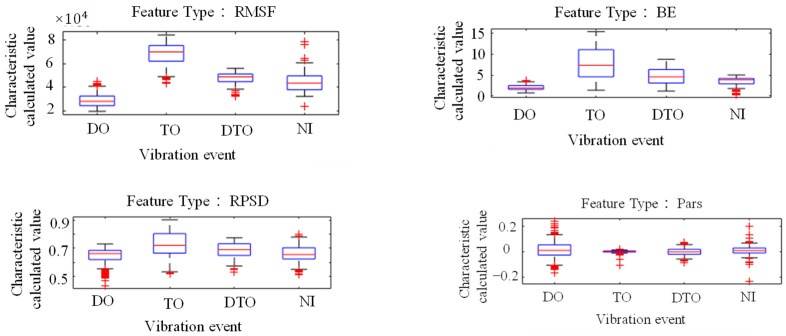
The 20 feature values of the four vibration events with 100% load intensity.

The tunnel and relied on the 20 features selected in Section 2.1; however, due to space limitations, that list includes only the feature data for the four vibration events with 100% load intensity. Therefore, the 20 feature values of the four vibration events with 100% load intensity are plotted in Figure 5. Based on the differences in the calculated values of the extracted features from the four vibration events, it can be seen that the feature PM is more discriminatory in terms of distinguishing tamping vibrations, the feature Var is more adept at identifying non-disturbance events, and the feature RMSF is more discriminatory in terms of distinguishing borehole vibration events.

To quantitatively assess the feature characteristics, the 20 features were assessed for variability via the one-way ANOVA method described in Section 2.1. The calculated values of the features were combined into a feature matrix, the label vector for the analysis of variance was obtained, and the calculated values are shown in Table 4. The calculated results show that the 20 features can effectively describe the variability of the events, and the 20 selected features can distinguish the four vibration events to some extent.

The 20 selected features can be effectively used as key indicators for classification and identification. However, strong correlations exist between some of the features, and these highly correlated features not only reduce the computational efficiency but also lead to an increase in the identification error for the subsequent classification model. Therefore, a correlation analysis of these 20 features is needed to identify and eliminate highly correlated features.

**Figure 6:**
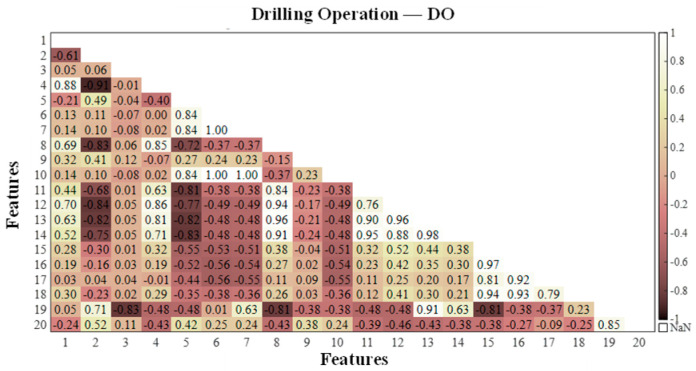
Correlation coefficients between different dynamic strain features related to drilling operations.

**Figure 7:**
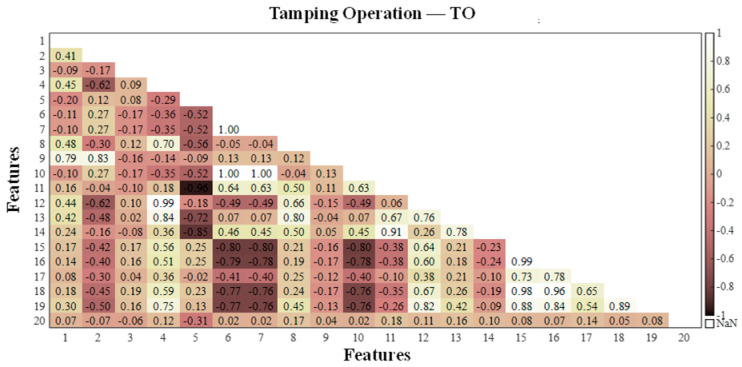
Correlation coefficients between different dynamic strain features related to tamping operations.

**Figure 8:**
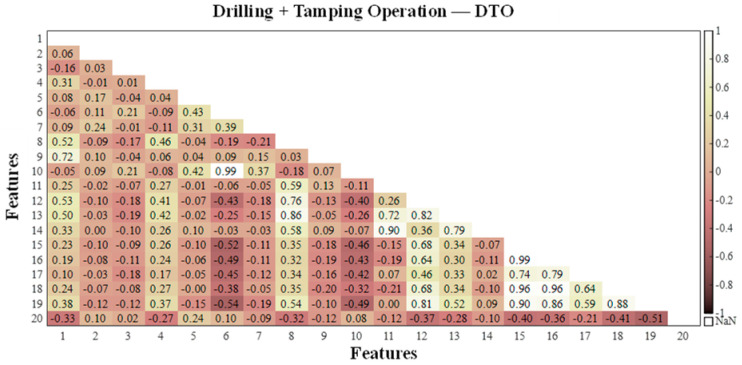
Correlation coefficients between different dynamic strain features related to drilling + tamping operations.

**Figure 9:**
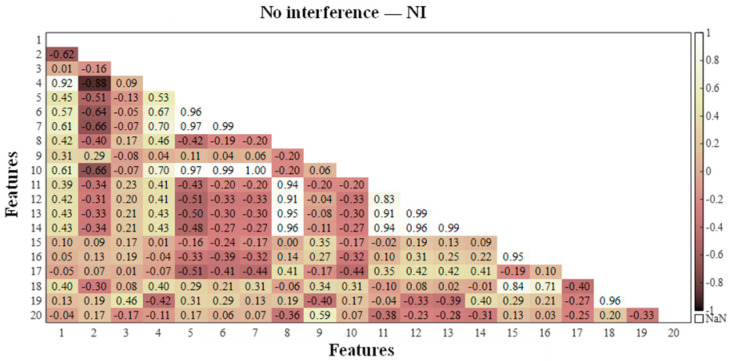
Correlation coefficients between different dynamic strain features with no interference.

According to the feature correlation analysis algorithm in Section 2.1, the feature matrix was constructed for the calculated values of each extracted working condition feature, and the correlation between the vibration event features was investigated via the Pearson correlation coefficient method. The Pearson correlation coefficients in the feature matrices of the four events were calculated, and to visually demonstrate the correlation between the features, a heatmap of the correlation coefficient matrix was generated, as shown in Figure 6, Figure 7, Figure 8 and Figure 9, where the horizontal and vertical coordinates represent the serial numbers of the 20 feature names.

By comprehensively studying the values of the correlation coefficients of different features in the four types of vibration events, the following evaluation results were obtained: among the four types of vibration events, there is a strong correlation between Var, Std and RMS; there is a high probability of a strong correlation between Sh, Cr, Im, CI and Kurt; and there is a certain probability of a strong correlation between CF, MSF, RMSF and BE. The highly correlated features as well as the similarly highly correlated features were eliminated, and five features, Var, Std, Sh, Kurt and CI, were discarded. Finally, the remaining 15 features were retained for the subsequent classifier training.

### 3.3. Results of Abnormal Vibrations in the Metro Protection Area

By employing the feature identification method based on dense measuring points and spatial correlation described in Section 2.2, the 15 features retained in each working condition data sample were updated. When the approach detailed in Section 2.2.2 was used to calculate the information for the dense measuring points, the information before and after the 15 measuring points was used for updating. When the distance-related confidence weights were calculated, the credibility decay coefficient was assumed to be −0.0025, and the credibility of the monitoring point information was assumed to be 0.75 when its own information was updated. While calculating distance-related confidence weights, the confidence attenuation coefficient was assumed to be −0.0025, and the information of the measurement point was updated by 0.75. When calculating the probability distribution-related confidence weights via kernel density estimation, the Epanechnikov kernel was selected for kernel density estimation, and the calculated values of the features for the 15 measurement points before and after the measurement point were used to estimate the probability distributions of the feature values.

**Figure 10:**
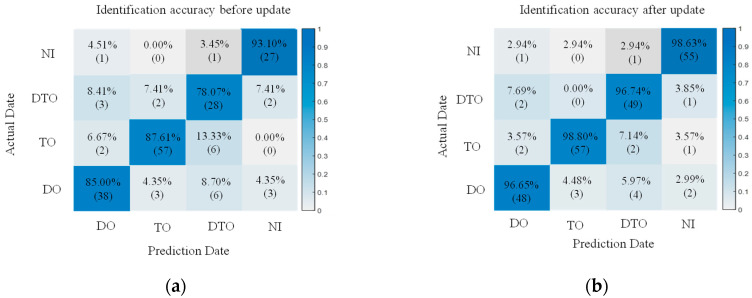
Confusion matrix of 100% load operation SVM identification. (**a**) SVM-I identification figure before the update. (**b**) SVM-II identification figure after the update.

**Figure 11:**
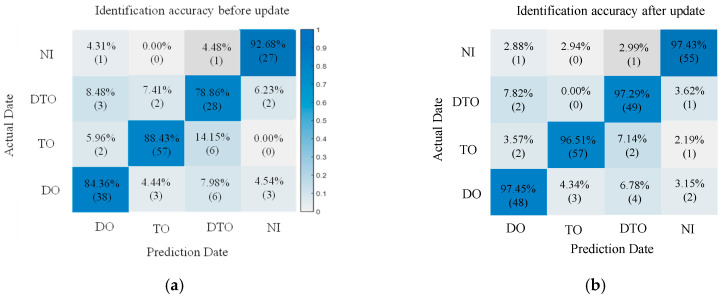
Confusion matrix of 100% load operation BP identification. (**a**) BP-I identification figure before the update; (**b**) BP-II identification figure after the update.

The 15 features under 50%, 100% and 200% vibration load intensities were used as the key features, and at the same time, according to the SVM and BP basic identification model constructed in Section 3.1, the identification effect of the classifier after the features were updated by the proposed method was preliminarily examined. The extracted updated feature values were used as the sample set, 75% of which were randomly selected as the training set, while the remaining 25% were used as the test set for training. The classification model without feature fusion updating is labeled I, and the classification model with feature fusion updating is labeled II. Moreover, a comparison group was created, and it adopted the un-updated feature values of these 15 features to train the model classifier. The trained model is denoted by model I. Due to space limitations, we list only the images from the SVM and BP identification results with 100% vibration load intensities, and the results of the training are shown in Figure 10 and Figure 11.

Based on the identification effects of the SVM and BP classification models in Figure 10 and Figure 11, it seems that both of them have higher identification accuracies and smaller differences in terms of the effects of identification. In addition, compared with other abnormal vibration activities, the joint activity of the DTO produces the greatest improvement effect in terms of the identification accuracy of operation simulation. This result is primarily due to the strong coupling effect of the DTO vibration signals, resulting in the existence of a modal overlapping phenomenon in the eigenspace for a single measurement point, and this interference makes it difficult for the classification model to effectively recognize abnormal vibrations in the mixed signals. After the eigenvalue fusion updating of the neighboring measurement points, the identification accuracy is greatly improved, indicating that the proposed method can overcome the limitations of traditional single-measurement point analysis when identifying multisource coupled signals and effectively improve the identification accuracy for abnormal vibrations in complex environments.

**Figure 12:**
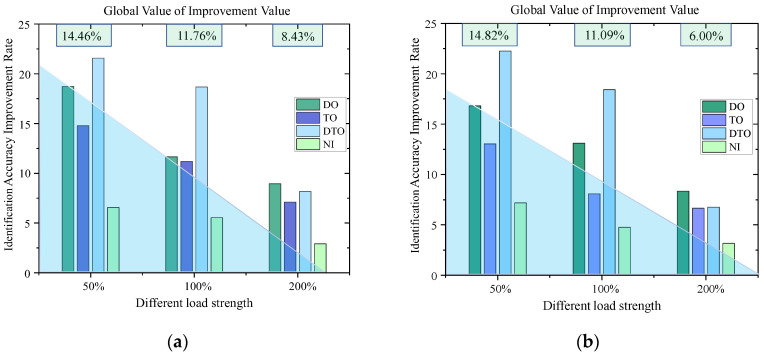
Identification accuracy improvement effect after update. (**a**) SVM Identification Accuracy Improvement Rate; (**b**) BP Identification Accuracy Improvement Rate.

The quantitative analysis of the experimental data in Figure 12 and Table 5 reveals that the identification accuracies for the features of the target measurement points are all improved after the fusion updating of the feature values for the neighboring measurement points. Under the high-load (200%) condition, without fusion updating of the neighboring measurement points, the identification accuracy is still high, indicating that the vibration signal-to-noise ratio triggered by the high-intensity load is significantly stronger than low-intensity load, and therefore, the abnormal vibration is a dominant feature in the time-frequency domain. Additionally, the classification model that does not rely on fusion updating also has a high identification rate. Under the weak-load (50%) condition, the identification accuracy improvement is best after fusion updating of the features from the target measurement point by the neighboring measurement point feature information.

With the gradual increase in load intensity from 50% to 200%, the identification accuracy of the SVM, BP and fusion-updated feature model increases nonlinearly. When the load intensity is 50%, the average identification enhancement rate of the three models can reach 14.5%, with the best increase in the identification accuracy; when the load intensity is 100%, the enhancement amplitude is reduced to 11.0%; and when the load intensity is 200%, the amplitude of the enhancement is reduced to 6.0–9.0%, and the enhancement effect of the identification accuracy is the smallest. Combined with the blue shaded area in Figure 12, the method proposed in this paper can effectively overcome the identification ambiguity problem caused by weak signals in the weak vibration stage after fusion updating by using the vibration information from the neighboring measurement points; however, this approach will cause the “marginal effect” related to feature fusion updating under high load intensities, which reduces the effect of the information from the neighboring points on the target point vibration incremental improvement for the features, leading to a decrease in the improvement for vibration features under high loading conditions.

## 4. Example of an Actual Tunnel

### 4.1. Extraction of the Dynamic Strain Features from Different Vibration Sources Generated in an Actual Metro Tunnel

To evaluate the vibration detection performance of the proposed method in a real-world setting, field experiments were carried out in an operational underground subway tunnel. A single-mode tight-buffered fiber optic cable with an outer diameter of 3 mm was deployed along the tunnel sidewall using a “trench-in-trench” installation method, forming a continuous distributed sensing network with a total length of 2 × 189 m.

Vibration signals were acquired using a distributed acoustic sensing (DAS) system, which functioned as both the laser pulse source and signal demodulation unit by emitting laser pulses into the sensing fiber and capturing the backscattered Rayleigh light. Key acquisition parameters were configured to ensure accurate characterization of subway-induced vibrations: the measurement range was set to 1000 m to fully cover the monitoring area with sufficient margin; the pulse repetition rate (PRR) was set to 10 kHz, corresponding to a Nyquist frequency of 5 kHz that adequately covers the dominant vibration frequencies of subway operations; and the spatial resolution was set to approximately 5 m, achieved with a 10-nanosecond pulse width, thereby defining each meter of fiber as an independent sensing unit. The system continuously recorded phase changes along the fiber at a sampling rate of 10 kHz, which are proportional to the dynamic strain (vibration).

**Figure 13:**
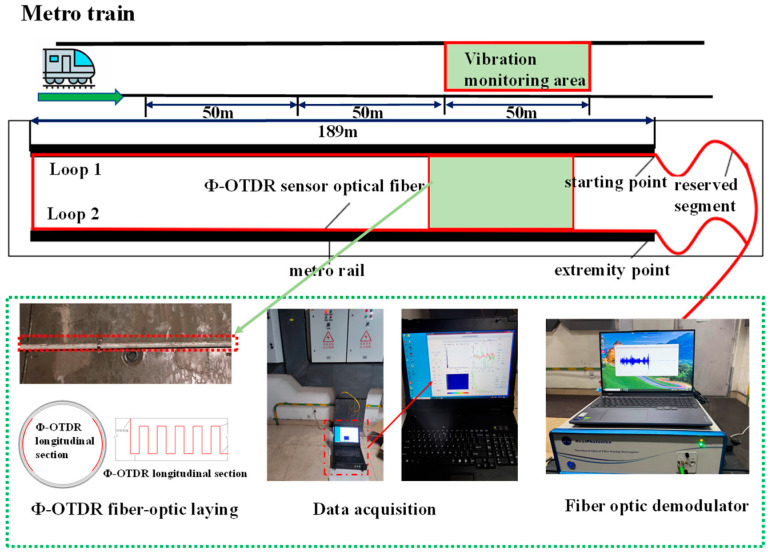
Design of the signal extraction scheme for different sources of vibrations.

**Figure 14:**
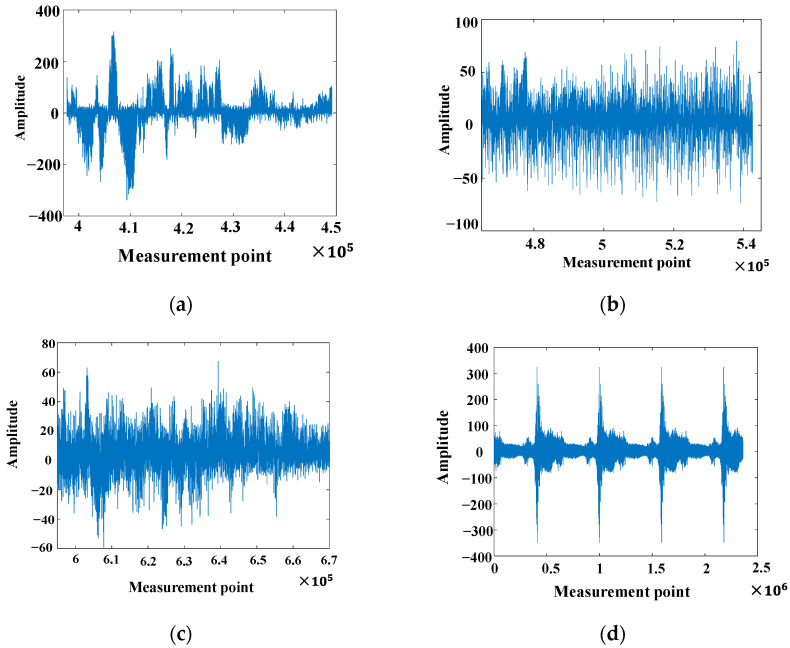
Dynamic strain signals from different sources of vibrations experienced by metro trains: (**a**) 0 m dynamic strain signal; (**b**) 50 m dynamic strain signal; (**c**) 100 m dynamic strain signal; (**d**) Dynamic strain signaling during train operation.

To validate the method under constrained fiber deployment conditions, a 50-m section near the tunnel midpoint was designated as the signal acquisition zone. Vibration tests were conducted with trains positioned at 100 m, 50 m, and 0 m from this zone. A baseline condition with no train operation was also included, forming four distinct vibration scenarios. The measured amplitude attenuation with distance within the acquisition zone was used to simulate the spatial response to different types of external anomalous construction activities. The specific experimental layout and data acquisition setup are illustrated in Figure 13 and Figure 14.

**Figure 15:**
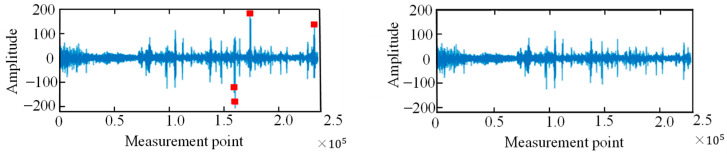
Handling of outliers (**left**: not handled; **right**: handled).

**Figure 16:**
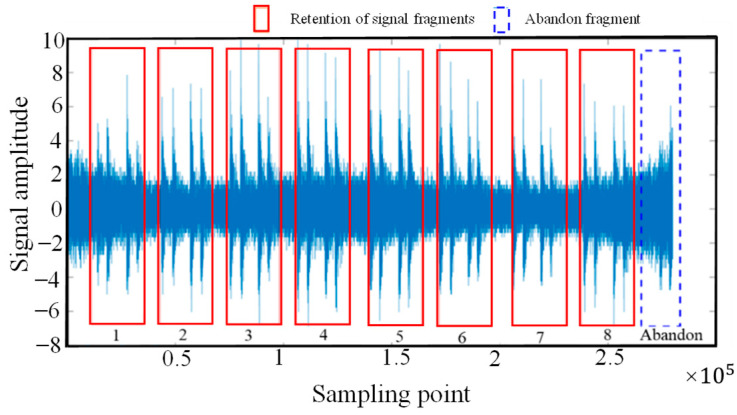
Vibration event signal after segmentation by endpoint detection.

The vibration signal data from φ-OTDR were automatically collected within 24 h on a certain day. Due to the characterization of the φ-OTDR system, the quality of the vibration sensor signal that was collected directly was generally relatively low; therefore, first, when the vibration signal preprocessing method mentioned in Section 2.1 was used, the collected φ-OTDR vibration data were preprocessed to eliminate outliers, and data centrality and standardization were completed. The data before and after data processing are shown in Figure 15. The vibration signals are segmented by the endpoint detection technique described in Section 2.1 and intercepted at different locations along the underground train according to their distance from the monitoring area, as shown in Figure 16.

**Figure 17:**
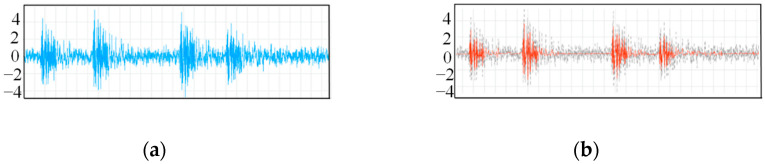
Comparison of the denoising effects of the coif wavelet basis (the gray region represents the original signal): (**a**) Original signal fragment; (**b**) Signal noise reduction processing of coif wavelet bases.

Although the acquired vibration signals were sliced through the data, the data obtained from the φ-OTDR acquisition system itself have non-smooth and nonlinear characteristics, and it was therefore necessary to conduct a denoising process to improve the data quality [39]. In this study, the coif (coiflet) wavelet technology was employed to process the vibration signals because it has good signal continuity and can yield significantly smaller noise signals. After completing data preprocessing, the collected signal and vibration signal before and after noise reduction is shown in Figure 17. A total of 620 groups of data were processed, and the details are shown in Table 6.

After data processing of the vibration signal, the 15 selected features after dimensionality reduction were extracted and calculated, and the 15-feature information retained by the experimental data samples under different working conditions was updated by the feature updating algorithm for vibration signals at dense measurement points, which is described in Section 2.3. Among them, 125 groups of feature calculation values under nighttime φ-OTDR of NI working conditions were used to construct the base identification model, assuming that abnormal vibration operations were found 35–55 m away from the monitoring area, and if no abnormal vibration was found during the identification process, the data baseline model that can be constructed is reasonable. In addition, the train distances from the signal acquisition area were 100 m, 50 m, and 0 m, with no train operation; as four different sources of abnormal vibration conditions were used, the vibration signals for those four conditions were collected, and features were extracted.

### 4.2. Results of Abnormal Vibrations in an Actual Tunnel

To comprehensively evaluate the effectiveness of the proposed method in terms of different classification models for real tunnels, different types of SVM and BP classification models were selected to identify the calculated vibration values at different training locations, and the performance of the models was comparatively analyzed and evaluated by using key indices such as the training time and accuracy rate.

When the SVM model was constructed, the PF and RBF kernels were selected as the kernel functions for training, and to compare and explore the effects of the two different kernel functions on the performance of the SVM classifier, the experimental design adopted a cross-validation method to evaluate the identification accuracy of the SVM classifier. During the training process for the implemented BP neural network, three classic optimization strategies, namely, gradient descent, stochastic gradient descent (SGD) and the Adam optimizer, were selected to provide a comprehensive measurement of the overall performance of the BP neural network classification model.

**Figure 18:**
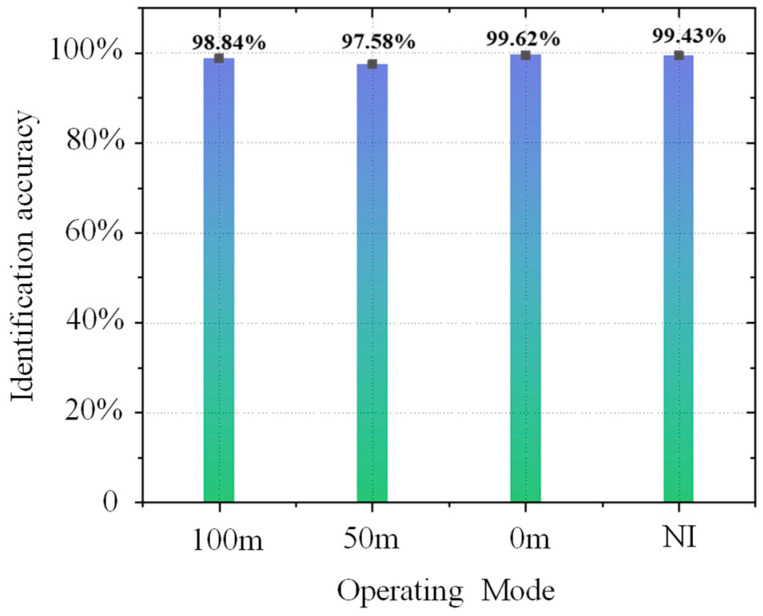
Abnormal operation identification accuracy at 35–55 m.

The calculated values of the four operating condition features were used as input data and introduced into the nighttime φ-OTDR detection and classification benchmark model described in Section 4.2. The different operating condition events were classified and detected, and the detection situation is shown in Table 7. Figure 18 shows the identification results assuming that there was a violation of construction work at 35–55 m. The abnormal disturbance identification is approximately 99% before and after updating, so it can be assumed that no abnormal vibration occurred in this area, and at the same time, the reasonableness of the construction of this experimental model is verified because it resulted in no misclassifications.

When identifying different vibration positions in subway trains in real tunnel projects, according to Table 7, the identification accuracy of the SVM classification model with the RBF kernel function is higher than that of the SVM classification model with the PF kernel function. After cross-validation, the identification accuracies of the SVM classification models with different kernel functions are improved, but the training time is significantly increased. This result indicates that the cross-validation method can improve the accuracy of the models to a certain extent when detecting vibration events in real engineering environments; however, it will increase the training time of the identification model.

The above seven classification models have high identification rates when recognizing vibration events in real engineering environments, with the SVM-type classification model having an identification accuracy of more than 85% and the BP neural network-type classification model having an identification accuracy of more than 95%. Among the BP neural network-based classification models, the classification model using the Adam optimization algorithm has the best identification effect. Therefore, the BP classification model using the Adam optimization algorithm is recommended for identifying abnormal vibrations in complex tunnel environments in actual projects.

According to the numerical simulation and actual tunnel experimental analysis, it can be seen that in the numerical simulation process, the identification accuracy of the SVM and BP neural network models and the identification accuracy after fusion updating are roughly comparable, which indicates that the two types of models are consistent with the response to feature optimization under the ideal conditions of low noise and linear predominance.

In an actual subway tunnel the experimental results show that the identification rate of the BP neural network is generally higher than that of the SVM; this phenomenon is due primarily to multisource interference (mechanical vibration, electromagnetic noise) in the measurement data, leading to difficulty when extracting the eigenvalues of the SVM classification model. Additionally, the BP model can be dynamically adjusted through the incremental learning of the weight matrix to achieve incremental compensation for nonlinear noise; therefore, it can gradually adapt to the state of the monitoring environment.

**Figure 19:**
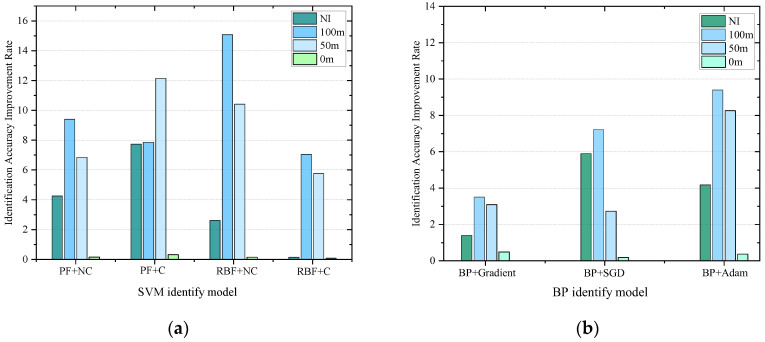
Identification accuracy improvement effect after update of different type classifier: (**a**) Identification effect of SVM model; (**b**) Identification effect of BP model.

After the fusion and updating of the vibration features by the proposed method, the identification accuracy of both the SVM and BP models are improved to a certain extent. Furthermore, through Figure 19 the identification accuracy improvement effect of the SVM is better than that of the BP neural network because, after feature fusion by the proposed method, performance compensation is realized for the weak model (SVM), which compensates for the nonlinear, large-scale and high-dimensional data; at the same time, the method has a double gain effect and optimizes the training convergence of the superior model (BP) to improve the identification accuracy.

## 5. Conclusions

To enhance the utilization of monitoring data between the dense measurement points of distributed sensing optical fiber applications in tunnels and achieve the precise identification of abnormal vibrations in protected metro areas, an identification method based on the spatial correlation of dynamic strain obtained from the dense measurement points of distributed sensing optical fibers is proposed. The following conclusions are drawn from the study:

(1) An accurate method for identifying abnormal vibrations in metro areas is proposed, and it uses the spatial correlation of dense measuring points to adjust the dynamic strain features of the target measuring points, thus improving the accuracy of identifying abnormal vibrations.

(2) The numerical results show that under weak-load operation, the identification accuracy improvement effect is best after fusion updating of the target measurement point features by the neighboring measurement point feature information, and the identification accuracies of the SVM and BP classification models are comparable under numerical simulation conditions; as the load intensity gradually increases, the identification accuracy of the classification model gradually improves, whereas the identification accuracy enhancement effect gradually decreases.

(3) The results of actual tunneling experiments show that the identification accuracy of the BP classification model is generally greater than that of the SVM in complex environments. After updating the feature fusion, the identification accuracy improvement effect of the SVM is better than that of the BP model, and the proposed method can effectively compensate for the limitations of the traditional classification model when handling complex nonlinear data.

(4) The BP classification model using the Adam optimization algorithm has the best identification effect, and its robustness in a dynamic noise environment is obviously better than that of the traditional model. It is recommended that the BP classification model using the Adam optimization algorithm be used to recognize abnormal vibrations in complex tunnel environments.

(5) The method is especially suitable for distributed optical fibers with dense measurement points. When the measurement points are sparse, it is necessary to supplement the information between the monitoring points to enhance the spatial correlation between the dense points.

## Figures and Tables

**Table 1:** Multidimensional time-frequency domain signals.

Ordinal Number	Feature Name	Feature Abbreviations	Ordinal Number	Feature Name	Feature Abbreviations
1	Maximum value	Max	11	Shape factor	Sh
2	Minimum value	Min	12	Crest factor	Cr
3	Mean	Me	13	Impulse factor	Im
4	Peak mean value	PM	14	Clearance factor	Cl
5	Average rectified value	Arv	15	Frequency of the center of gravity	CF
6	Variance	Var	16	Mean square frequency	MSF
7	Standard deviation	Std	17	Root mean square fluctuation	RMSF
8	Kurtosis	Kurt	18	Bandwidth energy	BE
9	Skewness	Skew	19	Relative power spectral entropy	RPSD
10	Root mean square	RMS	20	Peak average ratio	Pars

**Table 2:** Tunnel soil parameters.

Soil Layer Type	Serious (KN/m^3^)	Lateral Pressure Coefficient	Elasticity Modulus (MPa)	Poisson’s Ratio	Cohesive Force (KPa)	Internal Friction Angle (°)	Damping Ratio
Silty clay	19.6	0.42	9.8	0.40	12.0	35	4.2%
Sand	19.0	0.38	16.5	0.30	3.0	28	3.8%
Pebbly soil	21.5	0.28	45	0.21	0	35	1.5%
C50	24.5	0.25	3.45 × 10^4^	0.20	/	/	2.5%

**Table 3:** Peak vibration velocity of the tunnel lining at different locations under a ramming operation (mm/s).

Various Location	0	1/6π	1/3π	1/2π	2/3π	5/6π	π	7/6π	4/3π	3/2π	5/3π	11/6π	Total Value
0.5D	20.56	24.77	24.85	18.09	15.5	11.56	8.02	6.32	8.21	9.77	12.89	13.76	174.3
1.5D	22.54	25	21.82	17.33	12.8	12.34	7.57	5.86	8.15	11.36	12.87	16.69	174.33
3.0D	22.23	19.29	18.21	16.61	11.58	8.11	6.67	7.17	8.57	13.47	14.78	18.02	164.71

**Table 4:** χ value based on the results of the character variability analysis.

Feature Name	*χ*	Feature Name	*χ*	Feature Name	*χ*	Feature Name	*χ*
Max	6.28 × 10^−10^	Var	5.31 × 10^−10^	Sh	8.41 × 10^−14^	MSF	7.77 × 10^−8^
Min	4.13 × 10^−9^	Std	2.34 × 10^−10^	Cr	6.22 × 10^−9^	RMSF	1.29 × 10^−14^
Me	9.10 × 10^−3^	Kurt	1.32 × 10^−8^	Im	6.34 × 10^−18^	BE	9.08 × 10^−4^
PM	4.60 × 10^−11^	Skew	2.61 × 10^−13^	Cl	2.28 × 10^−20^	RPSD	1.86 × 10^−8^
Arv	5.49 × 10^−9^	RMS	2.14 × 10^−12^	CF	6.37 × 10^−9^	Pars	6.81 × 10^−3^

**Table 5:** Comparison of the SVM and BP identification accuracies before and after feature updating.

Loading Intensity	Classification Model	Identification Accuracy					
		DO	TO	DTO	NI	Global Value	Global Value of Improvement
50%	SVM-I	73.44%	83.46%	74.12%	89.43%	80.9%	14.63%
	SVM-II	92.16%	98.23%	95.69%	96.02%	95.53%	
100%	SVM-I	85.00%	87.61%	78.07%	93.10%	85.95%	11.76%
	SVM-II	96.65%	98.80%	96.74%	98.63%	97.71%	
200%	SVM-I	89.95%	92.61%	91.07%	97.10%	91.01%	8.43%
	SVM-II	98.89%	99.71%	99.23%	99.92%	99.44%	
50%	BP-I	71.68%	82.59%	75.33%	90.04%	79.91%	14.82%
	BP-II	88.49%	95.63%	97.58%	97.23%	94.73%	
100%	BP-I	84.36%	88.43%	78.86%	92.68%	86.08%	11.09%
	BP-II	97.45%	96.51%	97.29%	97.43%	97.17%	
200%	BP-I	90.12%	92.33%	92.41%	96.82%	92.92%	6.00%
	BP-II	98.46%	98.98%	99.15%	99.97%	98.92%	

**Table 6:** Experimental data collection.

Type of Vibration Signal	Metro Train Location Information			
Number of signal clusters	100 m	50 m	0 m	NI
	270	125	100	125

**Table 7:** Comparison of identification before and after updating of different classification models.

Type Classifier	Training Time (s)	Identification Accuracy of Different Vibration Sources			
		NI	100 m	50 m	0 m
SVM (PF kernel function + no cross validation)	Identification accuracy before update				
	157.5	92.18%	72.53%	74.36%	99.43%
	Identification accuracy after update				
	143.4	96.43%	81.92%	81.20%	99.58%
SVM (PF kernel function + cross validation)	Identification accuracy before update				
	277.2	89.96%	77.61%	72.48%	98.68%
	Identification accuracy after update				
	261.5	97.68%	85.46%	84.62%	98.98%
SVM (RBF kernel function + no cross validation)	Identification accuracy before update				
	98.6	95.98%	69.23%	72.48%	99.13%
	Identification accuracy after update				
	97.1	98.59%	84.31%	82.89%	99.27%
SVM (RBF kernel function) + cross-validation)	Identification accuracy before update				
	305.8	98.87%	80.52%	79.77%	99.35%
	Identification accuracy after update				
	286.3	99.00%	87.56%	85.52%	99.43%
BP Neural Network (Gradient descent optimization)	Identification accuracy before update				
	189.4	98.42%	93.45%	92.34%	99.33%
		Identification accuracy after update			
	165.5	99.83%	96.95%	95.43%	99.82%
BP Neural Network (SGD optimization)	Identification accuracy before update				
	143.2	92.89%	90.34%	92.44%	99.06%
	Identification accuracy after update				
	118.9	98.78%	97.55%	95.17%	99.24%
BP Neural Network (Adam optimization)	Identification accuracy before update				
	157.4	94.46%	89.41%	88.39%	99.15%
	Identification accuracy after update				
	124.2	98.63%	98.80%	96.65%	99.52%

## Data Availability

The data that support the findings of this study are available from Jinan Rail Transit Group Co., LTD but restrictions apply to the availability of these data, which were used under license for the current study, and so are not publicly available. Data are however available from the authors upon reasonable request and with permission of Jinan Rail Transit Group Co., LTD. Requests for data access should be directed to the corresponding author at 220146@sdjtu.edu.cn, who will facilitate the data-sharing process through formal collaboration agreements with Jinan Rail Transit Group Co., Ltd.
